# Calculation of Elastic Bond Constants in Atomistic Strain Analysis

**DOI:** 10.1186/s11671-015-1109-7

**Published:** 2015-10-16

**Authors:** Haiyuan Chen, Juanjuan Wang, Eric Ashalley, Handong Li, Xiaobin Niu

**Affiliations:** State Key Laboratory of Electronic Thin Film and Integrated Devices, University of Electronic Science and Technology of China, Chengdu, 610054 China; Institute of Fundamental and Frontier Sciences, University of Electronic Science and Technology of China, Chengdu, 610054 China

**Keywords:** Strain analysis, Poisson’s ratio, Elastic bond constant

## Abstract

Strain analysis has significance both for tailoring material properties and designing nanoscale devices. In particular, strain plays a vital role in engineering the growth thermodynamics and kinetics and is applicable for designing optoelectronic devices. In this paper, we present a methodology for establishing the relationship between elastic bond constants and measurable parameters, i.e., Poisson’s ratio *ν* and systematic elastic constant *K*. At the atomistic level, this approach is within the framework of linear elastic theory and encompasses the neighbor interactions when an atom is introduced to stress. Departing from the force equilibrium equations, the relationships between *ν*, *K*, and spring constants are successfully established. Both the two-dimensional (2D) square lattice and common three-dimensional (3D) structures are taken into account in the procedure for facilitating, bridging the gap between structural complexity and numerical experiments. A new direction for understanding the physical phenomena in strain engineering is established.

## Background

Strain always acts as a driving force in strain-engineered nanostructure formation [1–7] and plays a crucial role in nanoscale device designing. By manipulating strain-dependent growth kinetic and thermodynamic conditions, technically tailoring size, shape, and position of the growing structures becomes accessible. Accordingly, the energy band structures of the tailored structures can thus be modified. When shedding light on device designing, in particular, the strain-engineered formation of heterostructures, junctions, and variable composition profiles in quantum dots (QDs) and nanowires (NWs) during epitaxial growth [8, 9], strain provides a key strategy for producing optimal nanophotonic and nanoelectronic materials, including high-efficiency blue and green light-emitting diodes (LEDs) [10, 11], visible lasers [12–14], and high-efficiency solar cells [15]. Moreover, studies on the strain effect incorporated in two-dimensional (2D) materials [16–18] and topological insulators [19–21] also open doors to new classes of electronic and spintronic devices. Therefore, an understanding of the strain effects is highly essential.

Many efforts have been devoted to tackling the strain effects within epitaxial systems [22–29]. Among them, finite element (FE) method based on continuum elasticity and atomistic strain calculations are most commonly used. The FE method generally developed for macroscopic structures is integral to strain effect studies. However, to obtain accurate results, a smaller grid size is always favorable, which leads to increased computer memory and time. For the atomistic strain calculations, the bonds between atoms are often considered as ideal springs. All through the pioneering work by Keating [28] and other extended works, the empirical or semi-empirical interatomic potentials that are difficult to measure experimentally are always essential for the strain calculation. Thus, in the atomistic calculations, the lack of understanding of the elusive interaction coefficients may hinder better comprehension of the elastic properties.

In this work, we establish a connection between the Poisson’s ratio, a measurable elastic constant [30], and the spring constants used in the atomistic strain simulations, within the framework of linear elasticity theory. At the atomistic level, Poisson’s effect is caused by infinitesimal displacements of atoms thus the stretching of atomistic bonds within the material lattice to accommodate the stress. When the bonds in the stress direction are elongated or compressed, their counterpart in the perpendicular direction will be correspondingly shortened or lengthened. Drawing from the atomistic strain method [26], we considered the lattice bonds as ideal springs, connecting all the neighboring atoms and are within elastic deformation limit upon exerting external force. Our description of the elastic constants has a microscopic interpretation that incorporates all nearest and diagonal bond springs. Under the definition of Hooke’s law, the relationship between stress and responded strain is simply built, resulting in force balance equations, which is vital to formulating the relationship between the Poisson’s ratio and the spring constants.

## Methods

Focusing on deriving the spring constants in the atomistic strain method using the Poisson’s ratio, we give insights to building up the formulism for elasticity quantities based on the proposed approach. The explicit methodology presented here aims to develop a useful tool that can provide the inputs for atomistic strain analysis instead of a specific model. Drawing from the linear elasticity, the analytical results will facilitate one to directly use these inputs for most general crystal structures. Based on the implementation of our derivation, the validation of our formulated results is succeeded in our previous studies [27, 31, 32]; the elastic strain is well calculated utilizing our derived inputs. In the following derivation, both the common two-dimensional (2D) and three-dimensional (3D) crystal lattices are considered in our calculations. In comparison with the effect on atomistic strain analysis caused by nearest neighbors, some lower order quantities, like bond angle and nonlinear interaction, are ignored in our derivation. Although our result has its limitation when dealing with amorphous materials and systems with strong metallic bonds, it is constructed based on some general crystal lattices. It performs well in most materials with strong chemical bond. The derived explicit results are applicable to numerical calculation and computations, thus paving way for a more detailed exploration of strain-related mechanisms.

## Results and Discussion

### 2D Square Lattice

For a clear and easy to understand example of the general derivation to obtain the microscopic constants, we start with the 2D square lattice. The 2D square lattice is the most commonly used 2D simulation cell for qualitative studies of the general mechanisms. This simple generic 2D structure should capture the essential steps in the formulation and guide the study for more complicated systems.

**Fig. 1:**
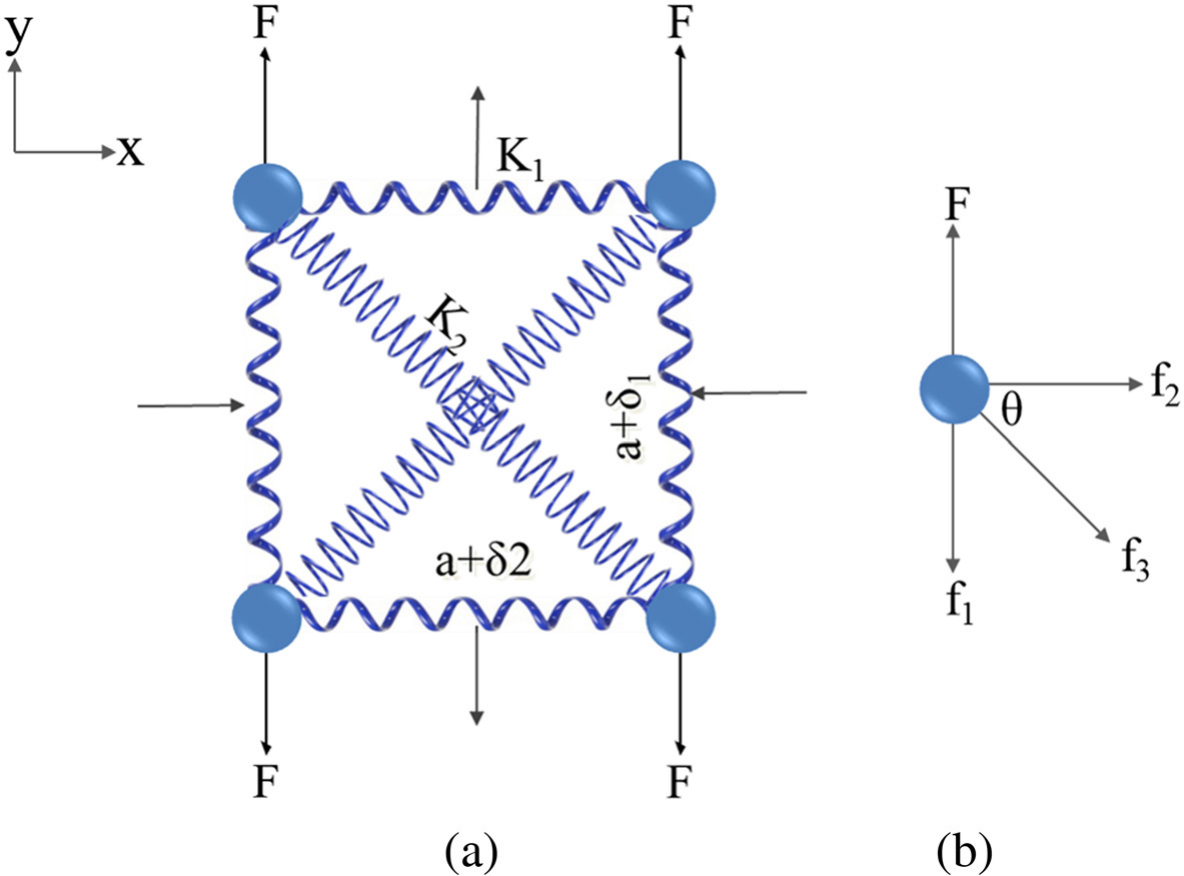
**a** A schematic illustration of 2D square system and **b** the selected atom (*upper left* in **a**) responded to forces

Assuming that the displacements of atoms, thus the infinitesimal changes of bond lengths, are along the axial direction, Poisson’s ratio *ν* is often expressed as *ν* = − *dε*_trans_/*dε*_axial_, where *ε*_trans_ and *ε*_axial_ represent the transverse and axial strains, respectively. The unit cell of a 2D square system demonstrated in Fig. 1a is composed of four atoms and has two types of spring bonds along the side and diagonal directions, respectively, with constants *K*_1_ and *K*_2_. By the Poisson’s ratio definition, when a stretching force *F* is exerted along the *y* direction, the resulted two length variations along *x* and *y* directions are expressed as *δ*_1_ and *δ*_2_, respectively, in which *a* represents the lattice constant and *δ*_1,2_ ≪ *a*. The change in diagonal length can be easily obtained by the simple trigonometric function. The total diagonal bond length is $\sqrt{{\left(a+{\delta}_1\right)}^2+{\left(a+{\delta}_2\right)}^2}$. Owing to the minuscule length variations, it can be approximately expressed as $\sqrt{{\left(a+{\delta}_1\right)}^2+{\left(a+{\delta}_2\right)}^2}\cong \sqrt{2}a+\frac{\delta_1+{\delta}_2}{\sqrt{2}}$ by ignoring the small second-order terms. Thus, the bond length change in the diagonal direction is described as ${\delta}_{\mathrm{diag}}=\left({\delta}_1+{\delta}_2\right)/\sqrt{2}$.

Next, we separate one atom (upper left in Fig. 1a) in this system and apply force balance analysis, shown in Fig. 1b. We get *f*_1_ + *f*_3_ sin *θ* = *F* and *f*_2_ + *f*_3_ cos *θ* = 0, in which *f*_1_ = *K*_1_*δ*_1_, *f*_2_ = *K*_1_*δ*_2_, and ${f}_3={K}_2{\delta}_{\mathrm{diag}}={K}_2\cdot \left({\delta}_1+{\delta}_2\right)/\sqrt{2}$ by Hooke’s law. Since *δ*_1,2_ ≪ *a*, *θ* ≈ 45^*°*^. Solving the above equations, we get the expression of Poisson’s ratio *ν* and the comprehensive elastic constant *K* of the whole system along the force direction

1 $$ \nu =-\frac{\delta_2}{\delta_1}=\frac{K_2}{2{K}_1+{K}_2}, $$

2 $$ K=\frac{F}{\delta_1}=\frac{2{K}_1\left({K}_1+{K}_2\right)}{\left(2{K}_1+{K}_2\right)}. $$

Note that both *ν* and *K* are measurable quantities in the lab. By solving the equation sets (1) and (2), finally, we get the relationship between *K*_1,2_, *K*, and *ν*

3a $$ {K}_1=\frac{K}{1+\nu}\kern0.5em , $$

3b $$ {K}_2=\frac{2\nu K}{1-{\nu}^2}. $$

Note that if *K*_2_ = 0, then *ν* = 0 and *K* = *K*_1_ as needed.

### Simple Cubic Lattice

**Fig. 2:**
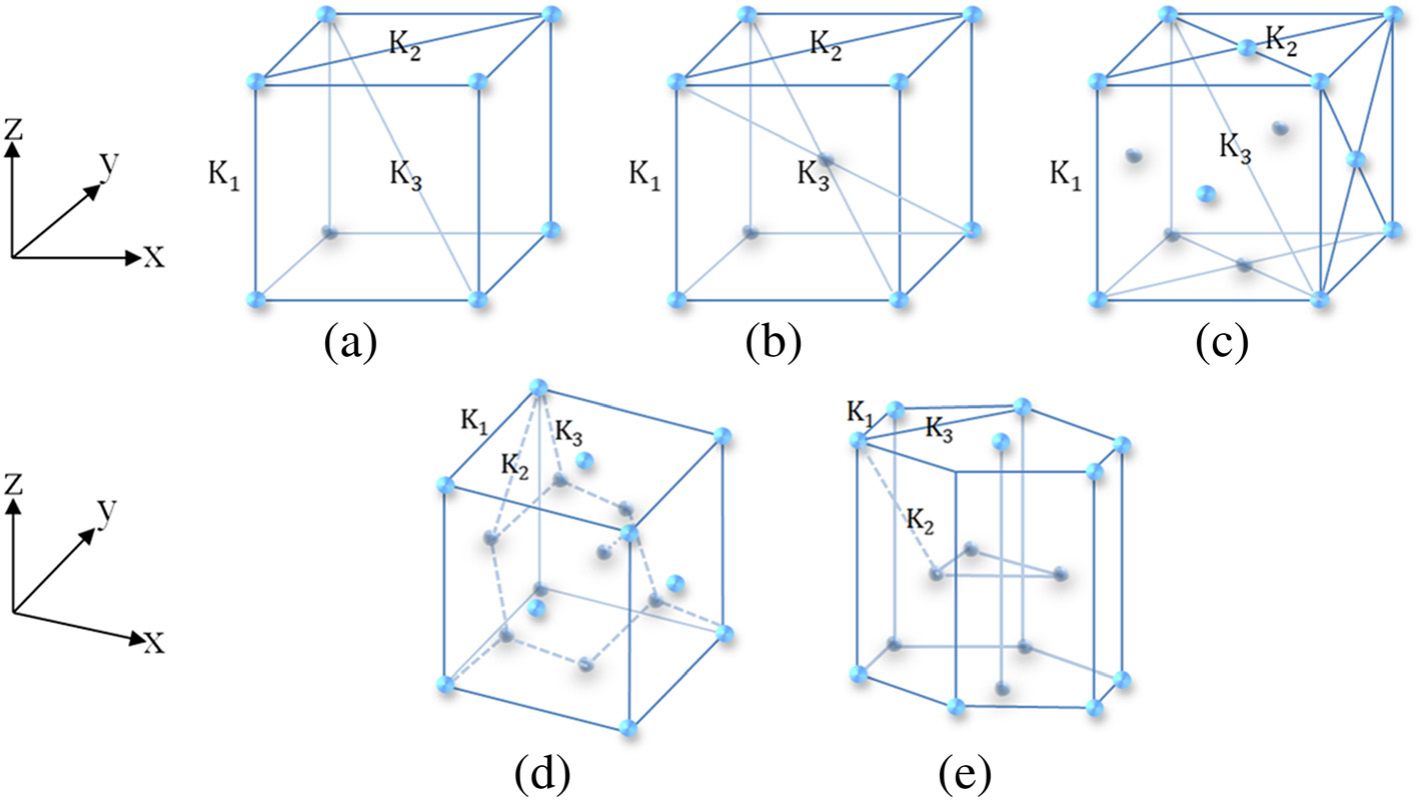
Schematic structures of **a** simple cubic, **b** body-centered cubic, **c** face-centered cubic, **d** diamond, and **e** hexagonal close-packed lattices

Having set up the 2D square lattice as a reference, we now derive the expressions of spring constants in terms of the Poisson’s ratio for common 3D crystal structures (shown in Fig. 2) following the established procedure.

We commence with choosing the simple cubic (SC) structure illustrated in Fig. 2a. We consider three distinct types of elastic springs along edge, face diagonal, and body diagonal directions, and the three main related coefficients are defined as *K*_1_, *K*_2_, and *K*_3_, respectively. Following the practice used for 2D cases, we set the displacement values *δ*_z_ = *δ*_1_ ≪ *a* and *δ*_x_ = *δ*_y_ = *δ*_2_ ≪ *a*, whiles the remaining values in the diagonal directions can be easily derived via geometry analysis.

From equilibrium conditions, the force balance equations are

4a $$ \left({K}_1+{K}_2+\frac{K_3}{3}\right){\delta}_1+\left({K}_2+\frac{2{K}_3}{3}\right){\delta}_2=F, $$

4b $$ \left(\frac{K_2}{2}+\frac{K_3}{3}\right){\delta}_1+\left({K}_1+\frac{3{K}_2}{2}+\frac{2{K}_3}{3}\right){\delta}_2=0. $$

According to Eq. (4b), the Poisson’s ratio is acquired through

5 $$ \nu =-\frac{\delta_2}{\delta_1}=\frac{3{K}_2+2{K}_3}{6{K}_1+9{K}_2+4{K}_3}. $$

Now, we substitute ${\delta}_2=-\frac{3{K}_2+2{K}_3}{6{K}_1+9{K}_2+4{K}_3}{\delta}_1$ into Eq. (4a), and the comprehensive elastic constant along *z*-axis is

6 $$ K=\frac{F}{\delta_1}=\frac{6{K_1}^2+6{K_2}^2+15{K}_1{K}_2+6{K}_1{K}_3+3{K}_2{K}_3}{6{K}_1+9{K}_2+4{K}_3}. $$

It is noticeable here that if *K*_2_ = 0, then $\nu =\frac{K_3}{3{K}_1+2{K}_3},K=\frac{3{K_1}^2+3{K}_1{K}_3}{3{K}_1+2{K}_3}$; if *K*_3_ = 0, then $\nu =\frac{K_2}{2{K}_1+3{K}_2},\ K=\frac{2{K_1}^2+2{K_2}^2+5{K}_1{K}_2}{2{K}_1+3{K}_2}$; and if *K*_2_ = *K*_3_ = 0, then *ν* = 0, *K* = *K*_1_ as required. Although *K*_1_, *K*_2_, and *K*_3_ are not attainable at the same time, the relationship can be used to set up the calculation inputs if one of the interactions is much weaker than the others.

### Body-Centered Cubic Lattice

Now, we pay attention to calculating the elastic constants of the body-centered cubic (BCC) lattice which is demonstrated in Fig. 2b.

Studying the balanced force conditions using the same definition of displacements, we have

7a $$ \left({K}_1+{K}_2+\frac{K_3}{6}\right){\delta}_1+\left({K}_2+\frac{K_3}{3}\right){\delta}_2=F $$

in *z* direction and

7b $$ \left(\frac{K_2}{2}+\frac{K_3}{6}\right){\delta}_1+\left({K}_1+\frac{3{K}_2}{2}+\frac{K_3}{3}\right){\delta}_2=0 $$

in *x* and *y* directions.

From Eq. (7b), we obtain the Poisson’s ratio,

8 $$ \nu =-\frac{\delta_2}{\delta_1}=\frac{3{K}_2+{K}_3}{6{K}_1+9{K}_2+2{K}_3}. $$

Substituting ${\delta}_2=-\frac{3{K}_2+{K}_3}{6{K}_1+9{K}_2+2{K}_3}{\delta}_1$ into the balanced condition Eq. (7a), the comprehensive elastic constant along *z* direction is described as

9 $$ K=\frac{F}{\delta_1}=\frac{12{K_1}^2+12{K_2}^2+30{K}_1{K}_2+6{K}_1{K}_3+3{K}_2{K}_3}{12{K}_1+18{K}_2+4{K}_3}. $$

It is observed here that if *K*_2_ = 0, then $\nu =\frac{K_3}{6{K}_1+2{K}_3},K=\frac{6{K_1}^2+3{K}_1{K}_3}{6{K}_1+2{K}_3}$; if *K*_3_ = 0, then $\nu =\frac{K_2}{2{K}_1+3{K}_2},\ K=\frac{2{K_1}^2+2{K_2}^2+5{K}_1{K}_2}{2{K}_1+3{K}_2}$; and if *K*_2_ = *K*_3_ = 0, then *ν* = 0, *K* = *K*_1_ as required. Finally, the connection between the Poisson’s ratio and elastic constant is established for BCC lattice.

### Face-Centered Cubic Lattice

Here, we will show insights into establishing the relationship between the Poisson’s ratio and the elastic constant of the face-centered cubic (FCC) lattice illustrated in Fig. 2c. Similarly, given the minor length changes in response to external force, we get the Poisson’s ratio by analyzing the force equilibrium conditions. The balanced equation in *z* direction is

10a $$ \left({K}_1+\frac{K_2}{2}+\frac{K_3}{3}\right){\delta}_1+\left(\frac{K_2}{2}+\frac{2{K}_3}{3}\right){\delta}_2=F, $$

and in *x* and *y* directions, the equation is given as

10b $$ \left(\frac{K_2}{4}+\frac{K_3}{3}\right){\delta}_1+\left({K}_1+\frac{3{K}_2}{4}+\frac{2{K}_3}{3}\right){\delta}_2=0. $$

And then, the Poisson’s ratio is expressed as

11 $$ \nu =-\frac{\delta_2}{\delta_1}=\frac{3{K}_2+4{K}_3}{12{K}_1+9{K}_2+8{K}_3}. $$

Then, we undertake substitution operations, replacing *δ*_2_ with ${\delta}_2=-\frac{3{K}_2+4{K}_3}{12{K}_1+9{K}_2+8{K}_3}{\delta}_1$ in Eq. (10a). Thus the comprehensive elastic constant along z axis is

12 $$ K=\frac{F}{\delta_1}=\frac{12{K_1}^2+3{K_2}^2+15{K}_1{K}_2+12{K}_1{K}_3+3{K}_2{K}_3}{12{K}_1+9{K}_2+8{K}_3}. $$

Here the observation about the relationship show that if *K*_2_ = 0, then $\nu =\frac{K_3}{3{K}_1+2{K}_3},\ K=\frac{3{K_1}^2+3{K}_1{K}_3}{3{K}_1+2{K}_3}$; if *K*_3_ = 0, then $\nu =\frac{K_2}{4{K}_1+3{K}_2},\ K=\frac{4{K_1}^2+{K_2}^2+5{K}_1{K}_2}{4{K}_1+3{K}_2}$, and if *K*_2_ = *K*_3_ = 0, then *ν* = 0, *K* = *K*_1_ as required. Here the related equations about Poisson’s ratio and elastic constant are built up.

### Diamond Lattice

Now we consider the elastic constants of diamond lattice generally adopted in many materials, including α-tin, the semiconductors silicon and germanium, and silicon/germanium alloys in any proportion. The diamond structure is sketched in Fig. 2(d), and we also define three various spring bond constants along the nearest bond directions, which are labeled as *K*_1_, *K*_2_, and *K*_3_ respectively. Apparently, the force balance equations are

13a $$ \left({K}_1+\frac{K_2}{2}+\frac{K_3}{12}\right){\delta}_1+\left(\frac{K_2}{2}+\frac{K_3}{6}\right){\delta}_2=F $$

in z direction, and

13b $$ \left(\frac{K_2}{2}+\frac{K_3}{6}\right){\delta}_1+\left({K}_1+\frac{3{K}_2}{2}+\frac{K_3}{3}\right){\delta}_2=0 $$

in x and y direction.

According to Eq. (13b) above, Poisson’s ratio is written as

14 $$ \nu =-\frac{\delta_2}{\delta_1}=\frac{3{K}_2+{K}_3}{12{K}_1+9{K}_2+2{K}_3}. $$

Next, we replace *δ*_2_ in Eq. (13a) with ${\delta}_2=-\frac{3{K}_2+{K}_3}{12{K}_1+9{K}_2+2{K}_3}$. After substituting, we obtain the comprehensive elastic constant along z direction

15 $$ K=\frac{F}{\delta_1}=\frac{48{K_1}^2+12{K_2}^2+60{K}_1{K}_2+12{K}_1{K}_3+3{K}_2{K}_3}{48{K}_1+36{K}_2+8{K}_3}. $$

It is recognized that if *K*_2_ = 0, then $\nu =\frac{K_3}{12{K}_1+2{K}_3},\ K=\frac{12{K_1}^2+3{K}_1{K}_3}{12{K}_1+2{K}_3}$, if *K*_3_ = 0, then $\nu =\frac{K_2}{4{K}_1+3{K}_2},\ K=\frac{4{K_1}^2+{K_2}^2+5{K}_1{K}_2}{4{K}_1+3{K}_2}$, and if *K*_2_ = *K*_3_ = 0, then *ν* = 0, *K* = *K*_1_ as required. Finally, we attain the relationship between Poisson’s ratio and elastic constant for diamond lattice.

### Hexagonal Close-Packed Lattice

We will concentrate on creating the formulation of Poisson’s ratio and elastic constants for hexagonal close-packed (HCP) lattice, which exist in many single element metals, such as Magnesium (Mg), Titanium (Ti), Hafnium (Hf), and Zinc (Zn). There are three different spring bond constants shown in Fig. 2. Here, we still chose Cartesian coordinates in order to facilitate the calculation.

In accordance with the same definition of tiny length changes, we obtain the force balance equations

16a $$ \left({K}_1+\frac{K_2}{2}+\frac{K_3}{9}\right){\delta}_1+\left(\frac{K_2}{2}+\frac{2{K}_3}{9}\right){\delta}_2=F $$

in *z* direction and

16b $$ \left(\frac{K_2}{4}+\frac{K_3}{9}\right){\delta}_1+\left({K}_1+\frac{3{K}_2}{4}+\frac{2{K}_3}{9}\right){\delta}_2=0 $$

in *x* and *y* directions.

Through Eq. (16b), we get the expression for the Poisson’s ratio

17 $$ \nu =-\frac{\delta_2}{\delta_1}=\frac{9{K}_2+4{K}_3}{36{K}_1+27{K}_2+8{K}_3}. $$

Now, we replace ${\delta}_2=-\frac{9{K}_2+4{K}_3}{36{K}_1+27{K}_2+8{K}_3}{\delta}_1$ in the balanced condition Eq. (16a), and then the comprehensive elastic constant along *z* direction is written as

18 $$ K=\frac{F}{\delta_1}=\frac{36{K_1}^2+9{K_2}^2+45{K}_1{K}_2+12{K}_1{K}_3+3{K}_2{K}_3}{36{K}_1+27{K}_2+8{K}_3}. $$

Notably, from the above equations, we see that if *K*_2_ = 0, then $\nu =\frac{K_3}{9{K}_1+2{K}_3},\ K=\frac{9{K_1}^2+3{K}_1{K}_3}{9{K}_1+2{K}_3}$; if *K*_3_ = 0, then $K=\frac{4{K_1}^2+{K_2}^2+5{K}_1{K}_2}{4{K}_1+3{K}_2}$; and if *K*_2_ = *K*_3_ = 0, then *ν* = 0, *K* = *K*_1_ as required.

Consequently, we have established the correlations between the Poisson’s ratio and spring bond constant for all the 3D systems exemplified in Fig. 2.

## Conclusions

We successfully proposed a methodology calculating the spring constants between neighbored atoms in atomistic strain analysis, using quantifiable physical quantities, i.e., Poisson’s ratio *ν* and comprehensive elastic constant *K*. This method describes the neighbored atom interactions with different spring bonds and gives insight into the force balance conditions, which contribute to obtaining the plausible results. The 2D square lattice and the ordinary 3D lattices are explicated. This derivation process gives a straightforward view of understanding the elastic constant connections in these systems and is evidentially useful for numerical calculation and computations. This will pave way for a more detailed exploration of strain-engineered nanostructure formation and functionalization of electronic devices.
